# Application of Artificial Intelligence in the diagnosis and treatment of colorectal cancer: a bibliometric analysis, 2004–2023

**DOI:** 10.3389/fonc.2024.1424044

**Published:** 2024-10-11

**Authors:** Lamei Sun, Rong Zhang, Yidan Gu, Lei Huang, Chunhui Jin

**Affiliations:** ^1^ Department of Oncology, Wuxi Hospital Affiliated to Nanjing University of Chinese Medicine, Wuxi, China; ^2^ Department of Traditional Chinese Medicine, Jiangyin Nanzha Community Health Service Center, Wuxi, China; ^3^ Department of General Surgery, Jiangyin Hospital Affiliated to Nanjing University of Chinese Medicine, Wuxi, China

**Keywords:** colorectal cancer, Artificial Intelligence, CiteSpace, VOSviewer, Bibliometrix, bibliometrics

## Abstract

**Background:**

An increasing number of studies have turned their lens to the application of Artificial Intelligence (AI) in the diagnosis and treatment of colorectal cancer (CRC).

**Objective:**

To clarify and visualize the basic situation, research hotspots, and development trends of AI in the diagnosis and treatment of CRC, and provide clues for research in the future.

**Methods:**

On January 31, 2024, the Web of Science Core Collection (WoSCC) database was searched to screen and export the relevant research published during 2004-2023, and Cite Space, VoSviewer, Bibliometrix were used to visualize the number of publications, countries (regions), institutions, journals, authors, citations, keywords, etc.

**Results:**

A total of 2715 pieces of literature were included. The number of publications grew slowly until the end of 2016, but rapidly after 2017, till to the peak of 798 in 2023. A total of 92 countries, 3997 organizations, and 15,667 authors were involved in this research. Chinese scholars released the highest number of publications, and the U.S. contributed the highest number of total citations. As to authors, MORI, YUICHI had the highest number of publications, and WANG, PU had the highest number of total citations. According to the analysis of citations and keywords, the current research hotspots are mainly related to “Colonoscopy”, “Polyp Segmentation”, “Digital Pathology”, “Radiomics”, “prognosis”.

**Conclusion:**

Research on the application of AI in the diagnosis and treatment of CRC has made significant progress and is flourishing across the world. Current research hotspots include AI-assisted early screening and diagnosis, pathology, and staging, and prognosis assessment, and future research is predicted to put weight on multimodal data fusion, personalized treatment, and drug development.

## 1 Introduction

Colorectal cancer (CRC) is a common malignant tumor of the gastrointestinal tract, and the global morbidity and mortality of CRC rank third and second among all cancers, respectively (1). According to Cancer incidence and mortality in China, 2022, China is inflicted with a high incidence of CRC, and its morbidity and mortality rank second and fourth among all malignant tumors, respectively (2). Contributors to CRC include gender, genetic factors, and family factors in addition to smoking, obesity, and poor lifestyle (3). Currently, CRC is usually diagnosed by laboratory tests, endoscopy, imaging, and histopathology, and treated with endoscopy, surgery, radiotherapy, chemotherapy, targeted therapy, and immunotherapy (4). Despite these diagnostic and treatment technologies, CRC is till prevalent in China, as shown by its continuous increase in morbidity and mortality in men during 2014-2018 (2). Artificial intelligence (AI) is revolutionizing the diagnosis and treatment of CRC, as shown by the advances in high-precision medical image analysis, endoscopic data processing, and digital pathology assessment, as well as personalized treatment and robot-assisted surgery. AI also help to better allocate and utilize medical resources and healthcare services (5–9).

Bibliometrics is a cross-cutting science that uses mathematical and statistical tools to quantitatively analyze scientific literature and provides standardized evaluation criteria to reduce subjective bias compared to traditional review studies (10). It can help researchers quickly understand the classic literature and core authors in a certain field as well as the research hotspots and development trends, in order to enhance the research efficiency and assist scientific research decisions (11, 12).

Recent years have witnessed an increasing number of studies related to the application of AI in CRC diagnosis. However, very few researchers have used bibliometric methods to systematically analyze studies in this area. In the only few bibliometric studies on related topics, they all focus on the analysis of the basic status of the research, without a detailed and extended analysis of the current research hotspots and future research trends (13, 14). Based on the Web of Science (WoS) core collection database, the present bibliometric study uses Cite Space (15), VoSviewer (16), Bibliometrix (17) to analyze the status quo, hotspots, and trends in the research of AI-related CRC diagnosis or treatment during 2004-2023, aiming to provide new ideas and clues for related research work.

## 2 Materials and methods

### 2.1 Data sources and search strategy

**Figure 1:**
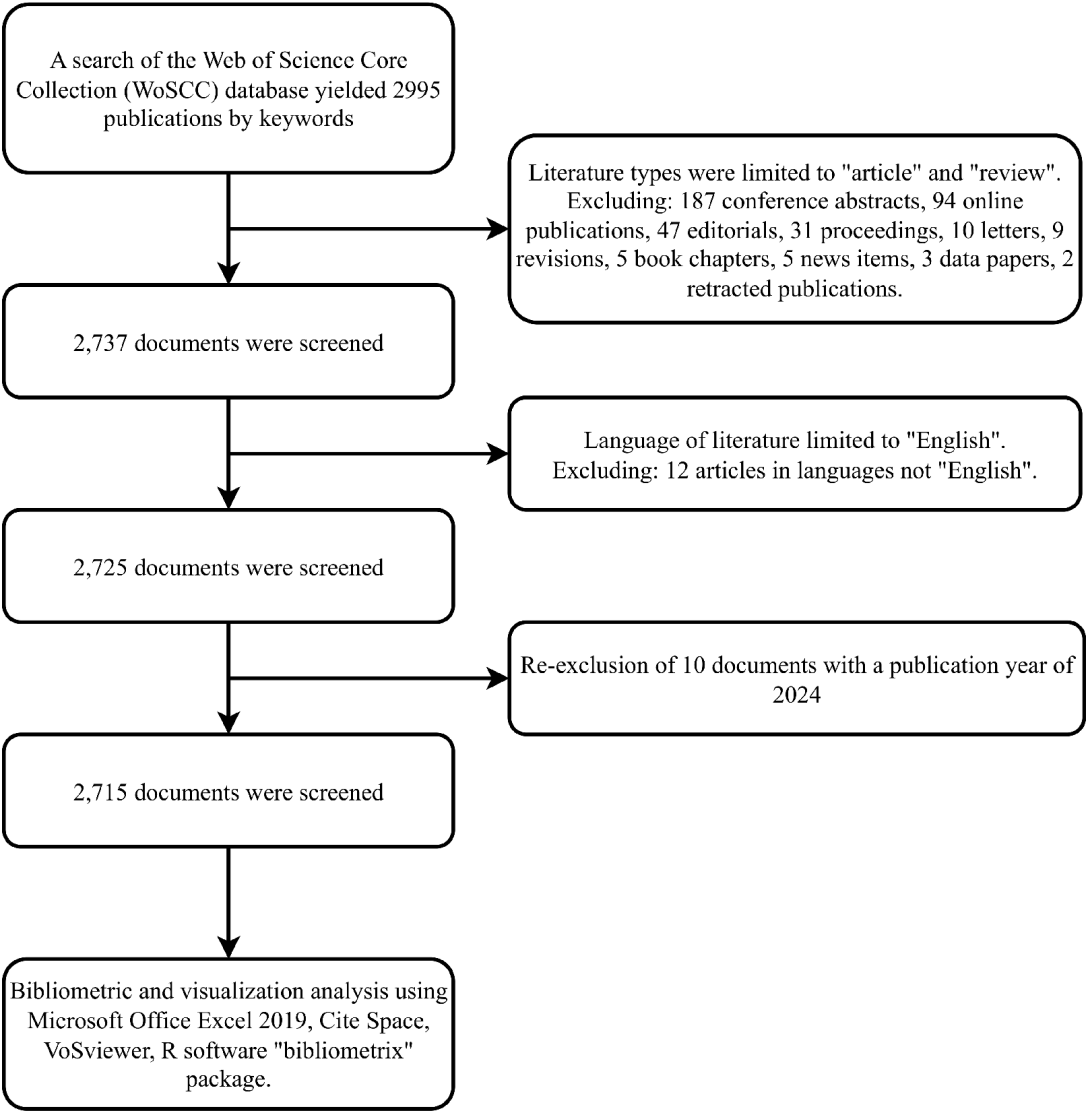
Flowchart of literature screening.

The WoS is the most comprehensive, systematic, and authoritative database available for bibliometric analysis, due to its rich bibliometric indicators and inclusion of high-quality journals from around the world (18). The data included in this study were obtained from the Web of Science Core Collection (WoSCC) database (https://www.webofscience.com/wos/woscc/basic-search). The search was conducted on January 31, 2024. Our search topic was divided into two parts, technical terms and disease terms, and for a broader search, we looked for relevant synonyms in the Pubmed MeSH Database and referred to recent bibliometric studies related to AI (19) or colorectal cancer (20) to finalize the following search formula: TS=(Rectal Neoplasm* OR Rectal Tumor* OR Rectal Cancer* OR Rectum Neoplasm* OR Rectum Cancer* OR Cancer of the Rectum OR Cancer of Rectum OR Colorectal Neoplasm* OR Colorectal Tumor* OR Colorectal Cancer* OR Colorectal Carcinoma* OR Colonic Neoplasm* OR Colon Neoplasm* OR Cancer of Colon OR Colon Cancer* OR Cancer of the Colon OR Colonic Cancer*) AND TS=(artificial intelligence OR Computational Intelligence OR machine learn* OR deep learn* OR artificial neural network OR machine intelligence).In the filtering function of the WoSCC database, we have set it so that only studies published as “article” and “review” and in “English” from January 1, 2004 to December 31, 2023 were screened. The process of literature screening is shown in **Figure 1**. A total of 2,715 articles were finally included, and the complete records of these articles (including title, authors, sources, abstracts, references, etc.) were exported in “download_txt” format for subsequent analysis.

### 2.2 Methods of statistical analysis

Microsoft Office Excel 2019 was used to draw the infographics of the annual publication volume and the descriptive tables of various types of data. VOSviewer (v.1.6.20) was used to carry out co-authorship and co-occurrence network analyses among countries (regions), institutions, authors, and journals. High-frequency co-cited literature, highly cited literature, and keywords were presented through network visualization, overlay visualization, and density visualization. Journal biplot overlays, co-cited literature clusters, keyword clusters, and timeline plots of keyword clusters were plotted using CiteSpace (v.6.3.R1 64-bit advanced) and combined with citation databases for literature analysis. Thematic trend analysis of keywords was run on the Bibliometrix (v4.1.4) based on R software (v4.3.2). The changes of research hotspots over time in this research area were visualized.

## 3 Results

### 3.1 General information about the data

A total of 2715 records were searched out of the WoS and imported into the Bibliometrix. The overall situation is shown in **Table 1**.

**Table 1:** Basic data information.

Description	Results
Documents	2715
Articles	2336
Reviews	379
Timespan	2004:2023
Sources (Journals, Books, etc)	722
Annual Growth Rate %	22.85
Document Average Age	3.41
Average citations per doc	18.74
References	89817

### 3.2 Analysis of the annual volume and its changing trend

**Figure 2:**
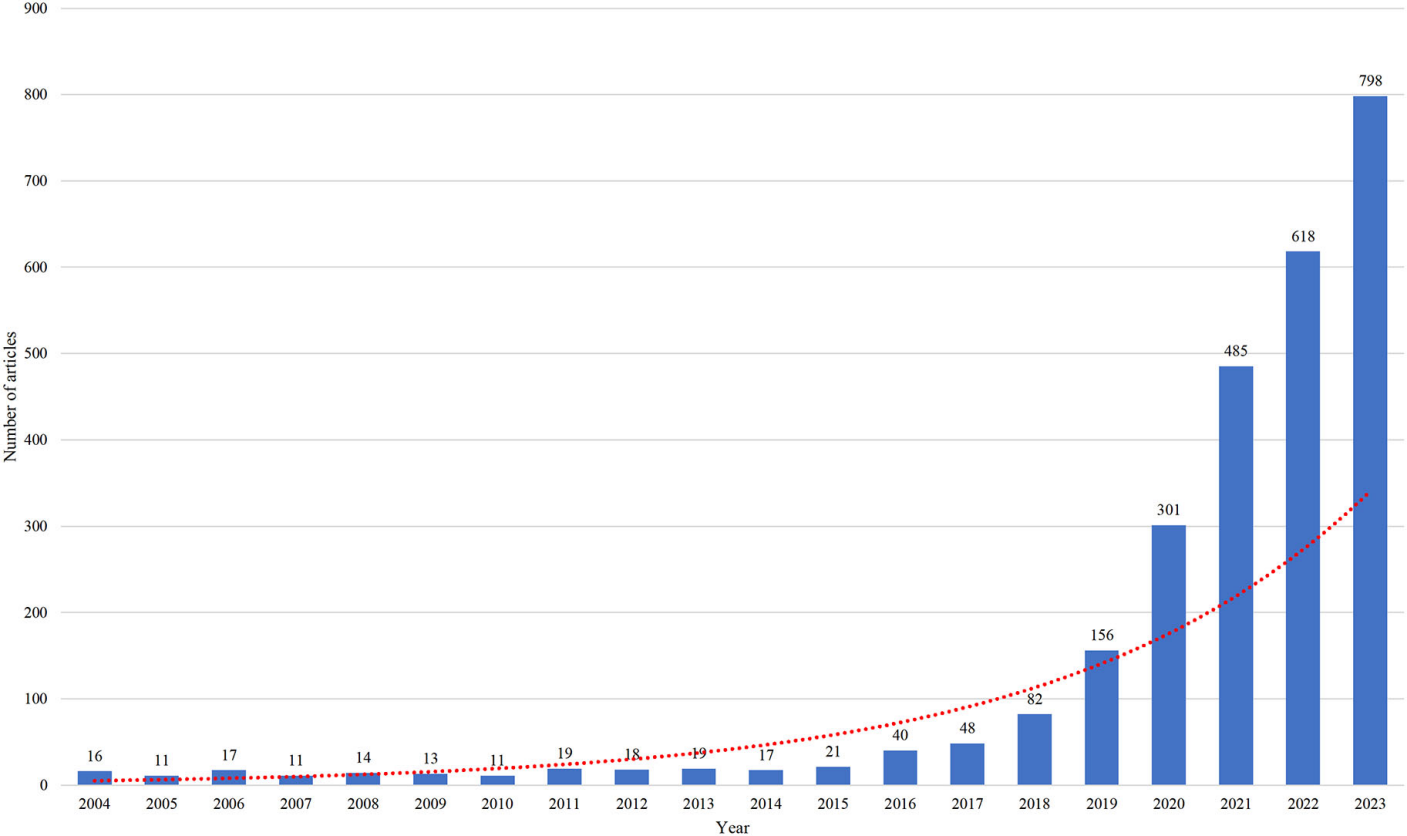
Trend in the annual number of articles published.

The annual numbers of articles were imported into Microsoft Office Excel 2019 to plot the trend of this index (**Figure 2**). It showed that the annual number of articles grew slowly, and never exceeded 50 before 2016, but rapidly after 2017, especially between 2020 and 2023, till to a peak of 798 in 2023.

### 3.3 Analysis of countries (regions)

The analysis of VOSviewer software showed that a total of 92 countries contributed to the research on AI in CRC diagnosis and treatment. The top 10 countries with the most publications are listed in **Table 2**, in which China ranked the first with 927 articles, the USA second with 658 articles, and the UK third with 240 articles. Although the USA took the second place in the total number of publications, its total number of citations reached 21,532 (the largest in the world) and average number of citations reached 32.7234, which indicates that the USA has a significant influence in this research field.

**Table 2:** Top 10 countries (regions) by number of publications.

Rank	Country	Documents	Percentage (%)	Citations	Avg. citations	TSL
1	China	927	34.14%	13550	14.617	320
2	USA	658	24.24%	21532	32.7234	647
3	United Kingdom	240	8.84%	7406	30.8583	393
4	Germany	204	7.51%	5336	26.1569	347
5	Italy	191	7.03%	3384	17.7173	292
6	Japan	188	6.92%	3506	18.6489	188
7	South Korea	163	6.00%	2318	14.2209	79
8	Netherlands	120	4.42%	3790	31.5833	272
9	India	119	4.38%	1022	8.5882	73
10	Canada	104	3.83%	2747	26.4135	156

TLS, Total link strength.

In addition, the average numbers of citations in the Netherlands (31.5833), the United Kingdom (30.8583), Canada (26.4153), and Germany (26.1569) were also relatively high, which may imply that the studies in these countries are generally of high quality. It is particularly noteworthy that although Sweden only ranked 17th in the number of publications (47 in total), its average number of citations was as high as 41.2128, which ranked the first among all the countries, implying that the quality of the research results in Sweden is high.

**Figure 3:**
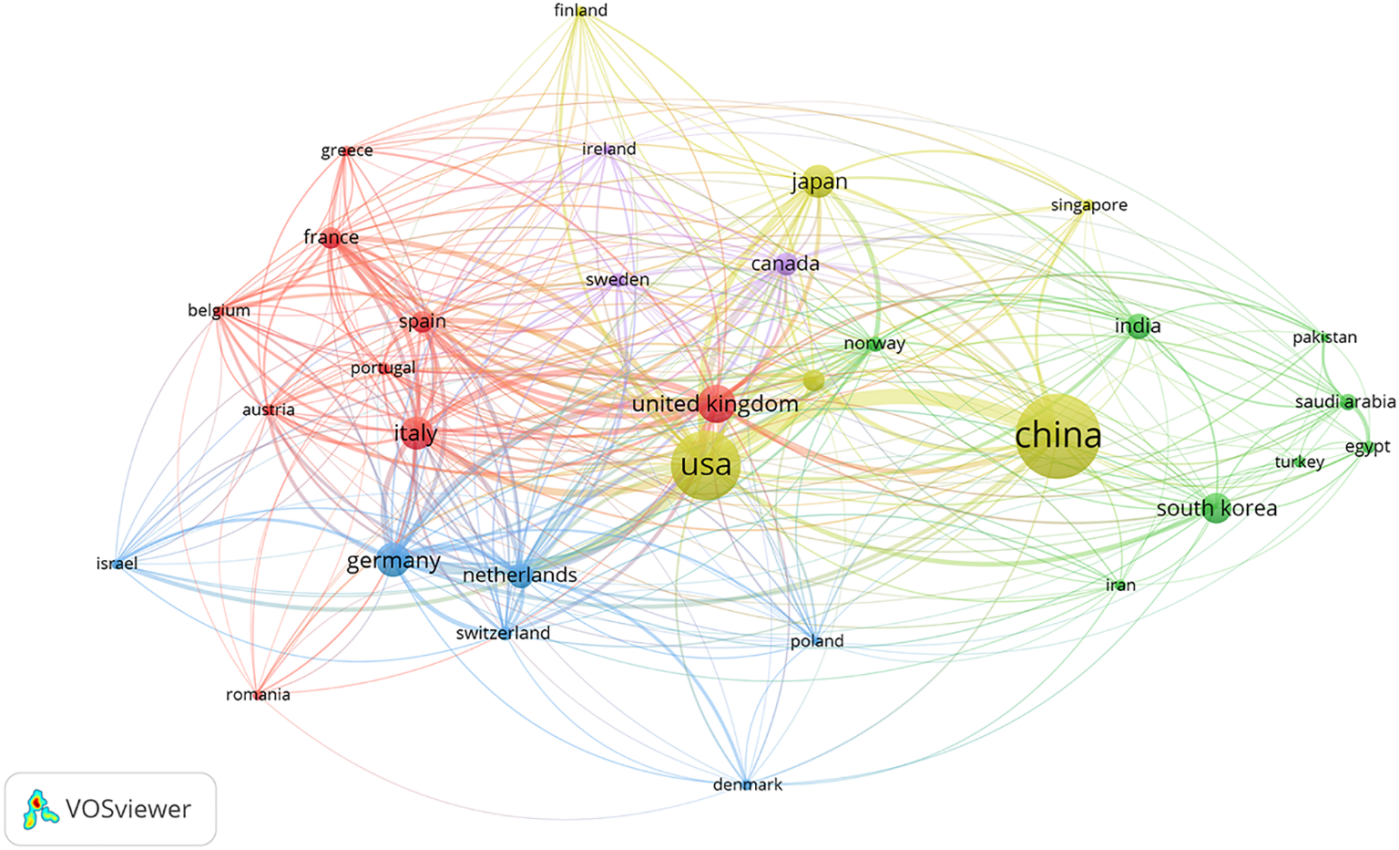
Networks of collaborations among countries (regions). Nodes indicate countries (regions); the size of nodes indicates the number of articles; a larger node indicates a larger number of articles; the color of nodes indicates the cluster to which the country (region) belongs; the line between nodes indicates the existence of an international collaboration; a thicker line indicates a closer collaboration.

Using the VOSviewer software, we performed a collaborative network mapping for the 32 countries with at least 20 publications (**Figure 3**), showing that international collaborations had appeared among a wide range of countries or regions in North America, Europe, and Asia, with the United States, the United Kingdom, Germany, China, and Italy as top centers.

### 3.4 Analysis of organizations

According to the results from the VOSviewer software, a total of 3,997 organizations have participated in the research on AI in CRC diagnosis and treatment. **Table 3** lists the top 10 organizations having published the most publications, in which Sun Yat-sen University ranked the first (70 articles), followed by Fudan University (53 articles), Chinese Academy of Sciences (51 articles), Harvard Medical School (50 articles), and Zhejiang University (50 articles). Among these top 10 institutions, Harvard Medical School had the most total citations (2,362), followed by the Chinese Academy of Sciences (1,707) and Sun Yat-sen University (1,313).

**Table 3:** Top 10 organizations in terms of number of articles issued.

Rank	Organization	Country	Documents	Citations	TSL
1	Sun Yat-sen Univ	China	70	1313	85
2	Fudan Univ	China	53	676	46
3	Chinese Acad Sci	China	51	1707	79
4	Harvard Med Sch	USA	50	2362	93
5	Zhejiang Univ	China	50	1186	11
6	Shanghai Jiao Tong Univ	China	48	460	45
7	Sichuan Univ	China	47	674	33
8	Southern Med Univ	China	46	595	52
9	Stanford Univ	USA	36	1574	29
10	Natl Canc Ctr	Italy	34	903	45

It is worth noting that although Mayo Clinic did not enter the top 10 in the number of publications (25), its total number of citations was as high as 3,662, topping all other institutions, indicating that its research quality was the highest.

**Figure 4:**
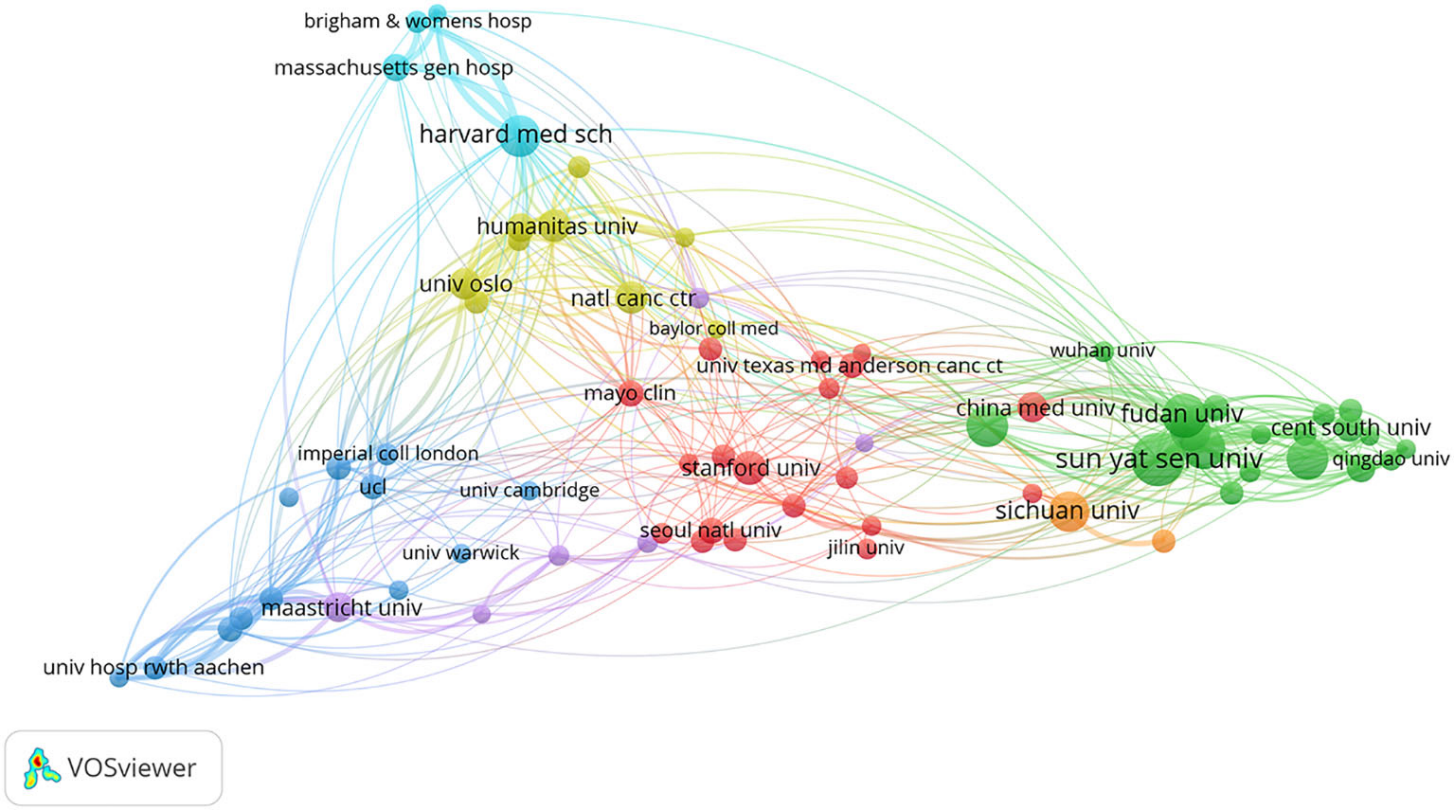
Mapping of inter-agency collaborative networks. Nodes represent organizations; the size of nodes indicates the number of publications; a larger node indicates a larger number of publications; the color of nodes indicates the cluster to which the organization belongs; the line between nodes indicates the existence of collaboration; a thicker line indicates a closer relationship.

In addition, a network involving 71 organizations having published at least 15 articles was constructed by the VOSviewer software (**Figure 4**). There were strong links between several institutions. The top five in TSL (Total Score Length) included the University of Oslo, Harvard Medical School, Sun Yat-sen University, Chinese Academy of Sciences, and Showa University, reflecting their leadership and influence in the research on AI in CRC diagnosis and treatment.

### 3.5 Analysis of authors

The results from the VOSviewer software showed that a total of 15,667 authors were involved in the research on AI in CRC diagnosis and treatment. As shown in **Table 4**, the top three authors with the most publications in this field were MORI, YUICHI (23), followed by REPICI, ALESSANDRO (20), and HASSAN, CESARE (18). As for the total number of citations, the top three authors were WANG, PU (10 articles with 1101 citations), BERZIN, TYLER M. (9 articles with 1088 citations) and KATHER, JAKOB NIKOLAS (17 articles with 1077 citations).

**Table 4:** Top 10 authors in terms of number of articles published.

Rank	Authors	Documents	Citations	TSL
1	MORI, YUICHI	23	758	164
2	REPICI, ALESSANDRO	20	701	108
3	HASSAN, CESARE	18	666	94
4	KATHER, JAKOB NIKOLAS	17	1077	45
5	KUDO, SHIN-EI	17	603	131
6	MISAWA, MASASHI	16	660	124
7	PICKHARDT, PERRY J.	15	436	25
8	MORI, KENSAKU	14	499	97
9	LIU, ZAIYI	13	147	54
10	SAITO, YUTAKA	13	425	55

**Figure 5:**
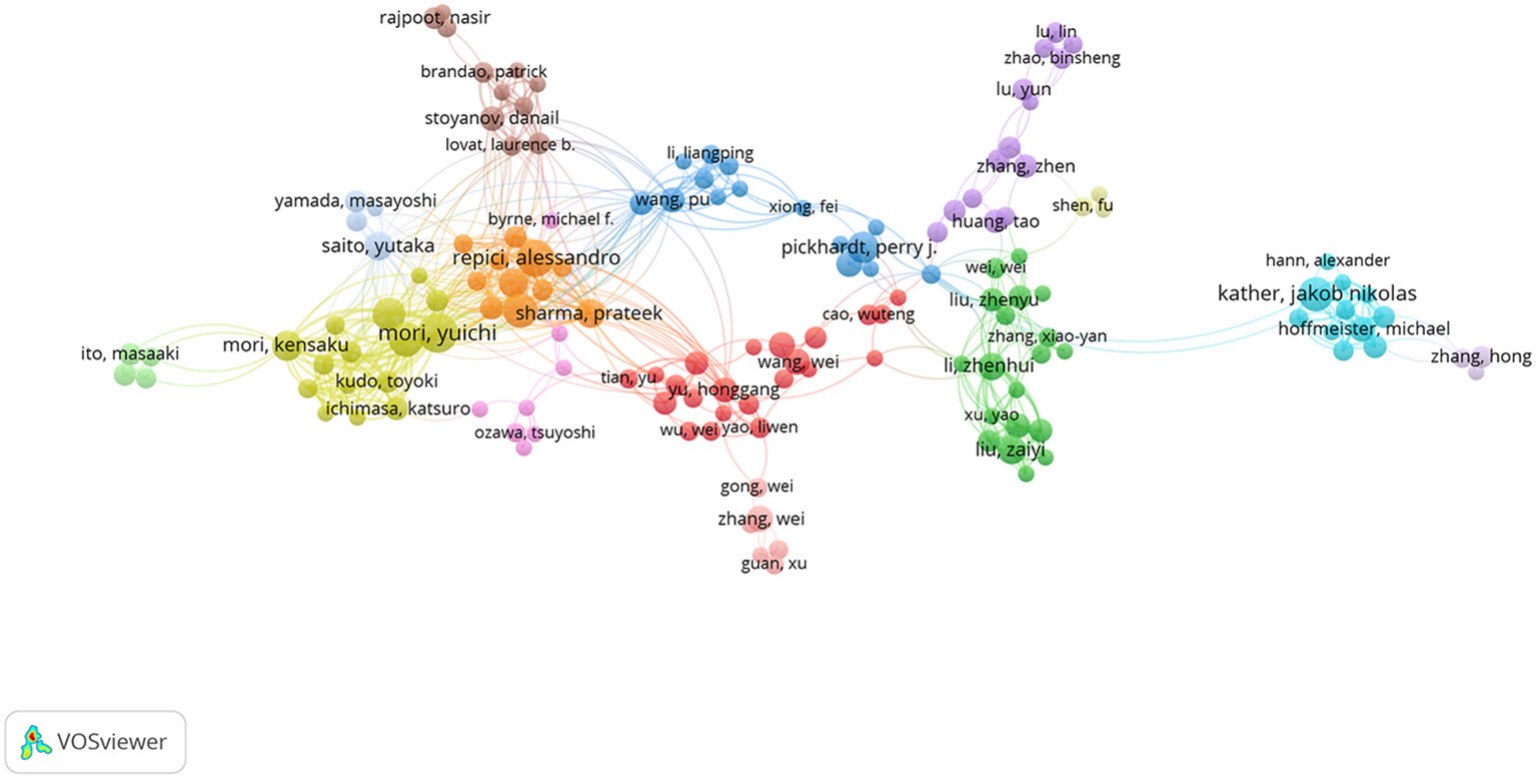
A network of inter-author collaborations. Nodes indicate authors; the size of nodes indicates the number of publications; a larger node indicates a larger number of publications; the color of nodes indicates the cluster of institutional authors; the line between nodes indicates the existence of an inter-author collaboration; a thicker line indicates a closer relationship.

In the VOSviewer software, a network of 234 authors with at least 5 publications was constructed (**Figure 5**), showing intense mutual collaborations between these authors. In this collaborative network, the top five authors in terms of TSL (Total Score Length) included MORI, YUICHI, KUDO, SHIN-EI, MISAWA, MASASHI, REPICI, ALESSANDRO, and MORI, KENSAKU, which indicated that they play more important roles in the collaborative network in this research area.

### 3.6 Analysis of journals and co-citations

Through the results from the VOSviewer software, we found that a total of 722 journals had published research on AI in CRC diagnosis and treatment. Among them, *Cancers* published the largest number of articles (110 articles). In terms of the number of citations, *IEEE Transactions On Medical Imaging* led with 3232 citations. **Table 5** provides details of the top 10 journals in the number of articles published, where *Computers In Biology And Medicine* carried the highest impact factor of 7.7 in 2022.

**Table 5:** Basic information of the top 10 journals in terms of number of publications and co-citations.

Rank	Journal	Documents	Citations	IF	JCR	Journal	Co-citations	IF	JCR
1	Cancers	110	1041	5.2	Q2	Gastrointest Endosc	2445	7.7	Q1
2	Frontiers In Oncology	99	578	4.7	Q2	Sci Rep-Uk	2288	4.6	Q2
3	Scientific Reports	81	2395	4.6	Q2	Gastroenterology	2246	29.4	Q1
4	Diagnostics	61	395	3.6	Q2	Nature	1618	64.8	Q1
5	Ieee Access	45	437	3.9	Q2	Plos One	1613	3.7	Q2
6	Medical Physics	44	1420	3.8	Q2	Gut	1582	24.5	Q1
7	Plos One	35	477	3.7	Q2	New Engl J Med	1542	158.5	Q1
8	WorldJournal Of Gastroenterology	35	360	4.3	Q2	J Clin Oncol	1493	45.4	Q1
9	Applied Sciences-Basel	30	490	2.7	Q3	Lect Notes Comput Sc	1426	——	——
10	Computers In Biology And Medicine	30	268	7.7	Q1	Endoscopy	1423	9.3	Q1

IF, Impact Factor (2022); JCR, Journal Citation Reports Subdivision (2022).

In terms of citation frequency, the top three journals were *Gastrointestinal Endoscopy* (2,445), *Scientific Reports* (2,288), and *Gastroenterology* (2,246), as shown in **Table 5**. Among these cited journals, *The New England Journal of Medicine* carried the highest impact factor of 158.5 in 2022.

**Figure 6:**
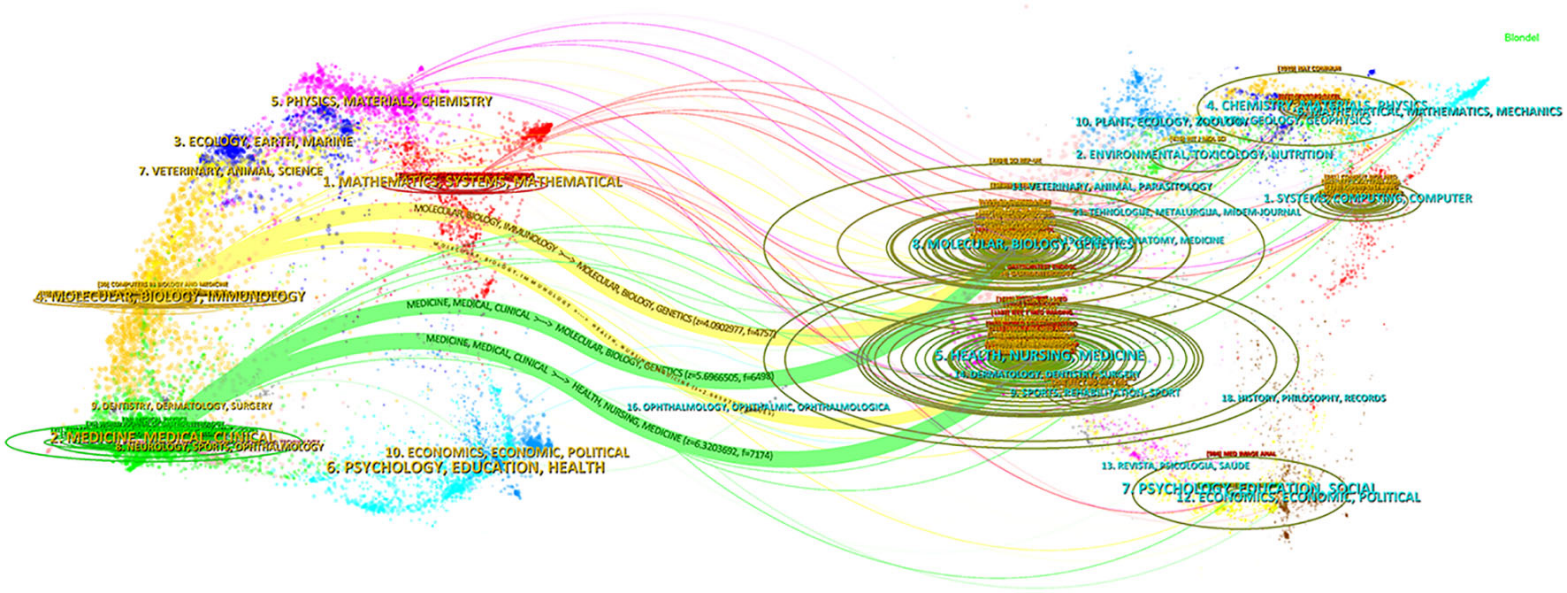
The dual-map overlay of journals. The left side is the cluster of citing journals and the right side is the cluster of cited journals; the curve in the middle is the citation curve; a longer vertical axis of the ellipse indicates that the journal publishes more papers, and a longer horizontal axis indicates a larger number of authors.

**Figure 6** shows a two-plot overlay mapping based on citing and cited journals in the CiteSpace software, revealing these journals were mainly cited through four paths: Literature published in journals in the fields of “Molecular, Biology, Genetics”, “Health, Nursing, Medicine” was cited by literature published in journals in the fields of “Medicine, Medical, Clinical”, “Molecular, Biology, Immunology”. This shows a complex network of intersections and influences between research areas.

### 3.7 Analysis of references

The analysis in the VOSviewer software showed that a total of 89,755 references were cited in 2715 articles. As shown in **Table 6**, the top 10 most cited literature included. In addition to three epidemiological studies on tumor incidence and mortality [Global cancer statistics 2018: GLOBOCAN estimates of incidence and mortality worldwide for 36 cancers in 185 countries (21), Cancer Statistics, 2017 (22), Global Cancer Statistics 2020: GLOBOCAN Estimates of Incidence and Mortality Worldwide for 36 Cancers in 185 Countries (1)], and a study examining the relationship between adenoma detection rate and CRC mortality risk [Adenoma Detection Rate and Risk of Colorectal Cancer and Death (23)], the remaining six pieces of literatures (24–29) focus on the application of convolutional neural networks in colonoscopy image recognition.

**Table 6:** Top 10 co-cited references.

Rank	Title	Co-citations	Year of publication
1	Global cancer statistics 2018: GLOBOCAN estimates of incidence and mortality worldwide for 36 cancers in 185 countries	269	2018
2	U-Net: Convolutional Networks for Biomedical Image Segmentation	203	2015
3	Deep Residual Learning for Image Recognition	186	2016
4	Cancer Statistics, 2017	168	2017
5	Very Deep Convolutional Networks for Large-Scale Image Recognition	151	2014
6	Real-time automatic detection system increases colonoscopic polyp and adenoma detection rates: A prospective randomized controlled study	134	2019
7	Global Cancer Statistics 2020: GLOBOCAN Estimates of Incidence and Mortality Worldwide for 36 Cancers in 185 Countries	131	2021
8	Adenoma Detection Rate and Risk of Colorectal Cancer and Death	126	2014
9	ImageNet classification with deep convolutional neural networks	125	2017
10	Deep learning localizes and identifies polyps in real-time with 96% accuracy in screening	115	2018

**Figure 7:**
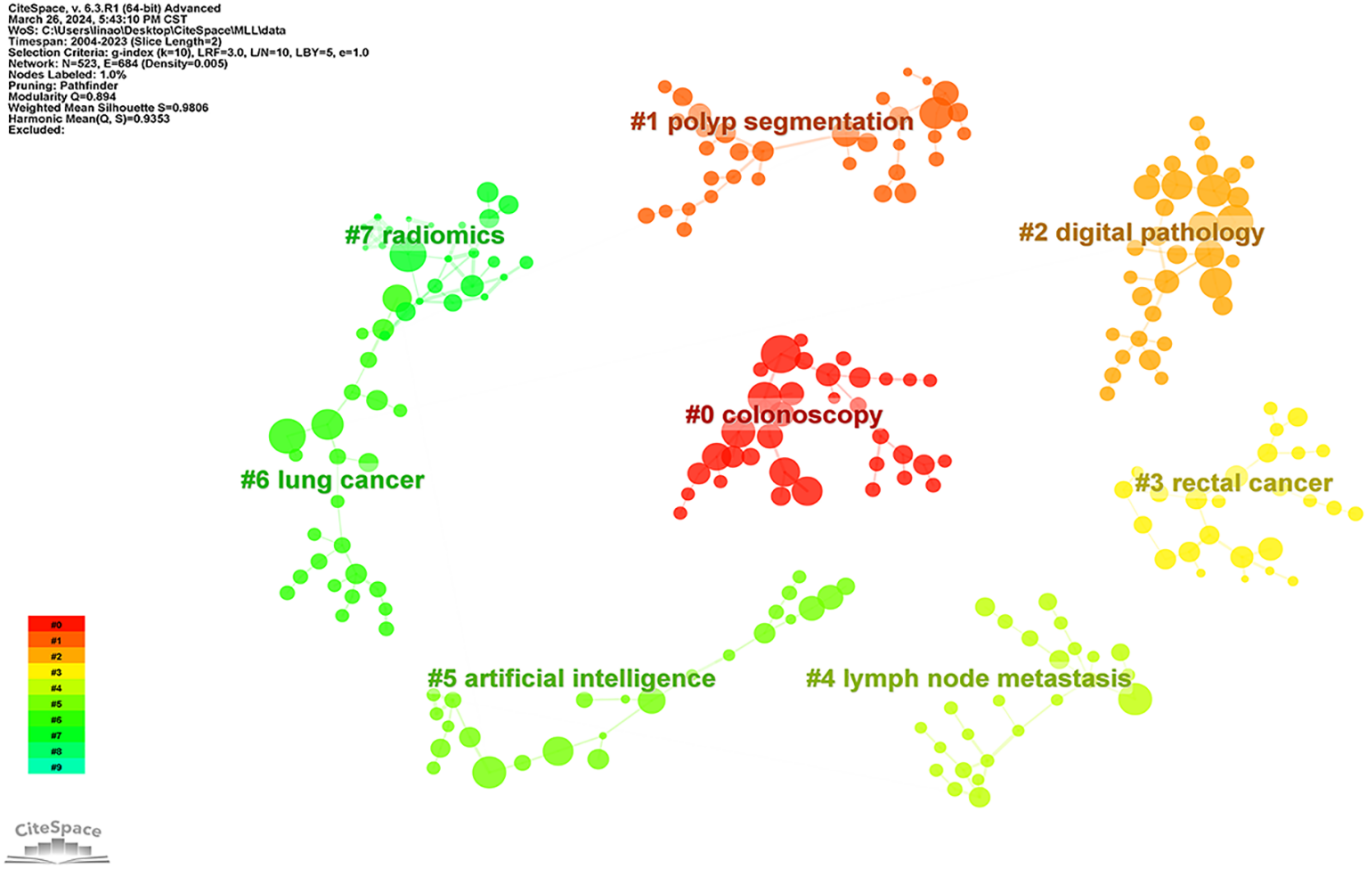
Clusters of co-cited references. Nodes indicate references; node size indicates the number of co-citations;, the larger the node indicates the higher the larger number of co-citations; the number indicates the number of the cluster, with smaller numbers indicating more references included in the cluster.

The co-cited literature was clustered and analyzed in Citespace software (**Figure 7**), where the major clusters were mainly related to “Colonoscopy”, “Polyp Segmentation”, “Digital Pathology”, “Lymph Node Metastasis” and “Radiomics “.

### 3.8 Analysis of highly cited studies

**Figure 8:**
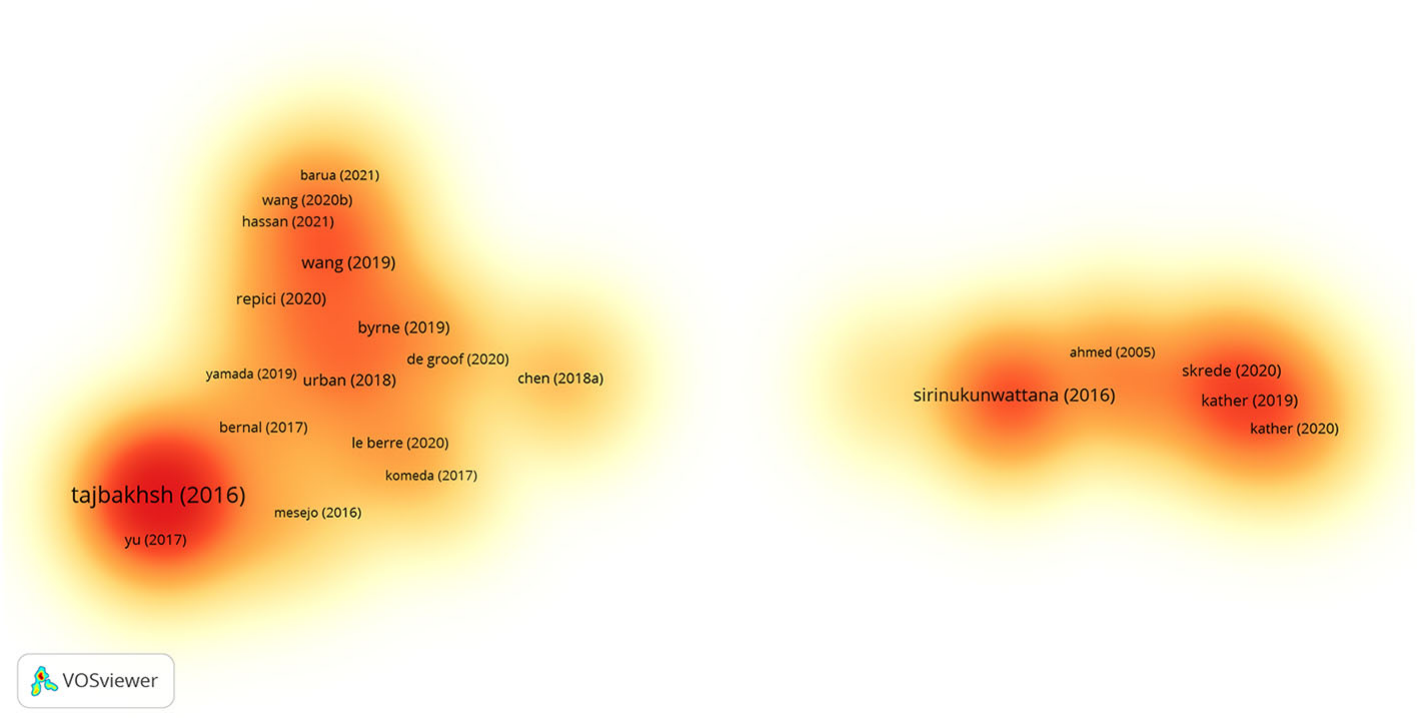
Documents citation density visualization. A color indicates the density of each publication. Yellow indicates a lower frequency of occurrence, while orange indicates a higher frequency of occurrence.

Highly cited studies are often regarded as authoritative in the field of research, as they provide key evidence and information on research progresses and trends. In 2715 pieces of results from the VOSviewer software, a total of 83 studies with more than 100 citations were screened out, of which 37 were strong interconnections, and their density is visualized in **Figure 8**. **Table 7** shows the top 10 cited literature, which were published mainly between 2016 and 2019.

**Table 7:** Top 10 cited publications.

Rank	Title	Citations	Year of publication
1	Convolutional Neural Networks for Medical Image Analysis: Full Training or Fine Tuning?	1687	2016
2	Locality Sensitive Deep Learning for Detection and Classification of Nuclei in Routine Colon Cancer Histology Images	713	2016
3	Genomic and Molecular Landscape of DNA Damage Repair Deficiency across The Cancer Genome Atlas	620	2018
4	Genome-wide cell-free DNA fragmentation in patients with cancer	561	2019
5	The Applications of Radiomics in Precision Diagnosis and Treatment of Oncology: Opportunities and Challenges	437	2019
6	Real-time automatic detection system increases colonoscopic polyp and adenoma detection rates: a prospective randomized controlled study	412	2019
7	Predicting survival from colorectal cancer histology slides using deep learning: A retrospective multicenter study	388	2019
8	Deep Learning Localizes and Identifies Polyps in Real Time With 96% Accuracy in Screening Colonoscopy	383	2018
9	Real-time differentiation of adenomatous and hyperplastic diminutive colorectal polyps during analysis of unaltered videos of standard colonoscopy using a deep learning model	358	2017
10	Deep learning-based tissue analysis predicts outcomes in colorectal cancer	334	2018

Among these highly cited studies, an article by TAJBAKHSH N et al. (30) published in *IEEE Transactions on Medical Imaging*, entitled “Convolutional Neural Networks for Medical Image Analysis: Full Training or Fine Tuning?”, received the largest number of 1687 citations. This article discussed how to more effectively apply convolutional neural networks to medical image analysis. The second most cited was an article by SIRINUKUNWATTANA K et al. (31) in the *IEEE Transactions on Medical Imaging* entitled “Locality Sensitive Deep Learning for Detection and Classification of Nuclei in Routine Colon Cancer Histology Images”, with 713 citations. It described the use of deep learning techniques to assist in the diagnosis and staging of CRC. Another representative study was “Genomic and Molecular Landscape of DNA Damage Repair Deficiency across The Cancer Genome Atlas” published by KNIJNENBURG TA et al. (32) in *Cell Reports*, cited for 620 times. This study involved the application of AI in identifying tumor gene expression patterns.

Among the top 10 cited studies, three focused on diagnostic colonoscopy, two on medical image analysis, two on genomic studies, two on prognostic analysis, and one on exploration for digital pathology. This broad coverage highlights the diverse and active research in this field.

### 3.9 Keywords analysis

#### 3.9.1 Keyword co-occurrence analysis

**Figure 9:**
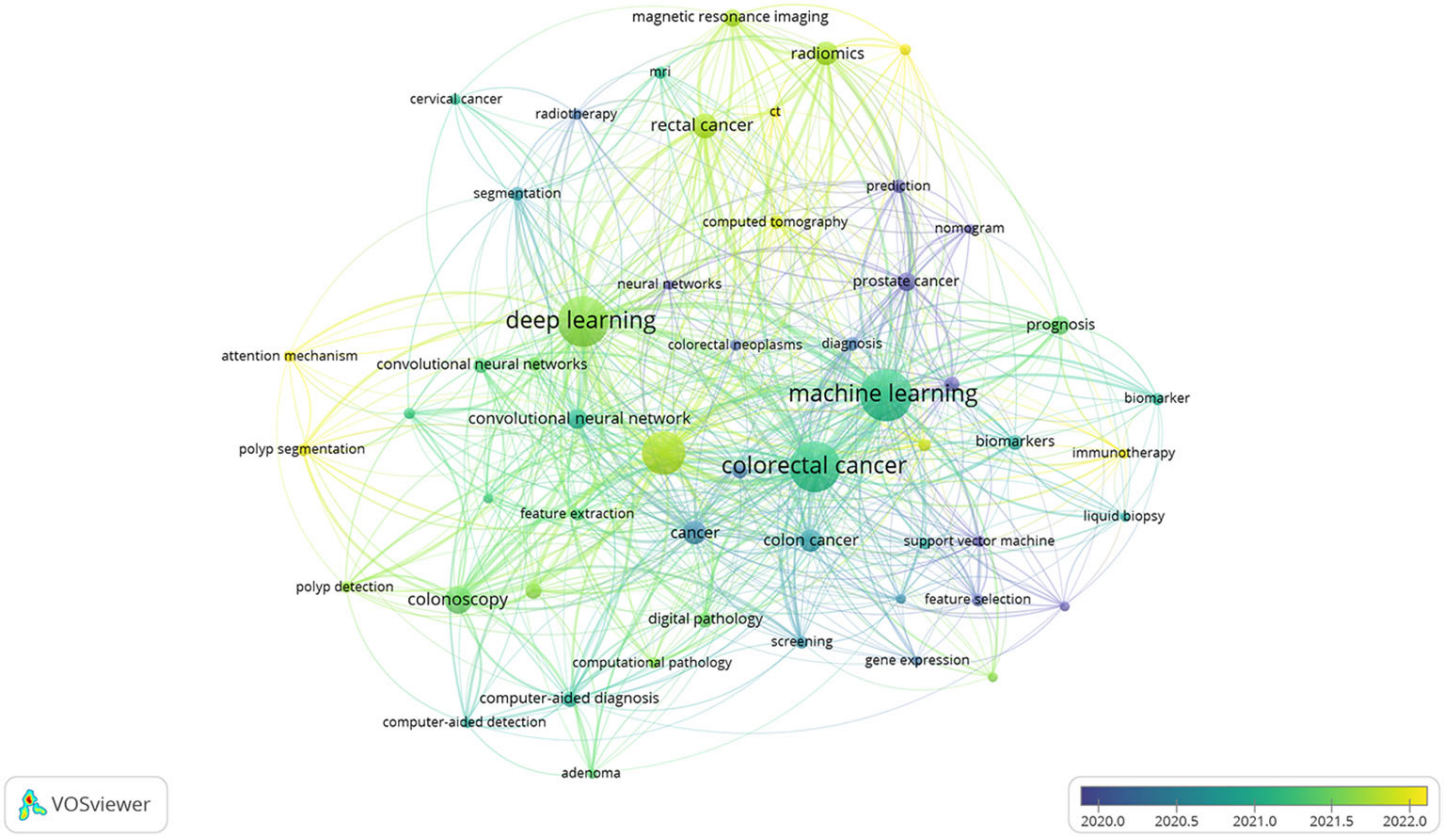
Keyword co-occurrence time overlay. Nodes indicate keywords; a node size indicates the frequency of one keyword; a larger node indicates a higher frequency of one keyword; the color of nodes indicates the time of keyword appearance; a color closer to blue indicates the earlier appearance of the keyword; a color closer to yellow indicates the later appearance of the keyword; the line between the nodes indicates the co-occurrence relationship between the keywords; a thicker line indicates a closer relationship.

Co-occurrence of these keywords can reveal the most active topics within a research field. After importing the data into the VOSviewer software, the results showed a total of 4882 keywords. The top 20 keywords with the highest frequency were displayed (**Table 8**). The keywords that appeared more than 500 times included machine learning, deep learning, and CRC. Then, the popular keywords were categorized according to the type and functionality of AI models used in these studies (**Table 9**). The top five types of AI models were machine learning, deep learning, convolutional neural networks, artificial neural networks, and transfer learning; while in terms of functionality, the top five were colonoscopy, radiomics, prognosis, magnetic resonance imaging, and endoscopy. In addition, the VOSviewer software was used to plot the 54 keywords with more than 20 occurrences into a time-stacked graph (**Figure 9**), which demonstrated the evolution of keywords over time. As shown by gradation of colors in the graph, a color closer to yellow indicates that this keyword is more recent. As can be seen from the timeline in the bottom right corner of the figure, the top keywords were more concentrated between 2020 and 2022. Relatively new keywords included computed tomography, attention mechanism, immunotherapy, lymph node metastasis, and polyp segmentation. The concentrations of these keywords reflected the latest trends and developments in the research.

**Table 8:** Top 20 keywords in terms of frequency of occurrence.

Rank	keywords	Occurrences	Rank	keywords	Occurrences
1	machine learning	547	11	prostate cancer	76
2	deep learning	545	12	prognosis	70
3	colorectal cancer	528	13	Magnetic resonance imaging	61
4	artificial intelligence	399	14	endoscopy	56
5	colonoscopy	156	15	classification	48
6	rectal cancer	136	16	artificial neural network	46
7	convolutional neural network	131	17	computer-aided diagnosis	46
8	radiomics	121	18	biomarkers	45
9	cancer	116	19	digital pathology	45
10	colon cancer	109	20	segmentation	44

**Table 9:** Classification of hot keywords.

classification	keywords	Occurrences	classification	keywords	Occurrences
model	machine learning	547	function	colonoscopy	156
	deep learning	545		radiomics	121
	convolutional neural network	131		prognosis	70
	artificial neural network	46		Magnetic resonance imaging	61
	transfer learning	43		endoscopy	56

#### 3.9.2 Keyword clustering and theme trend analysis

**Figure 10:**
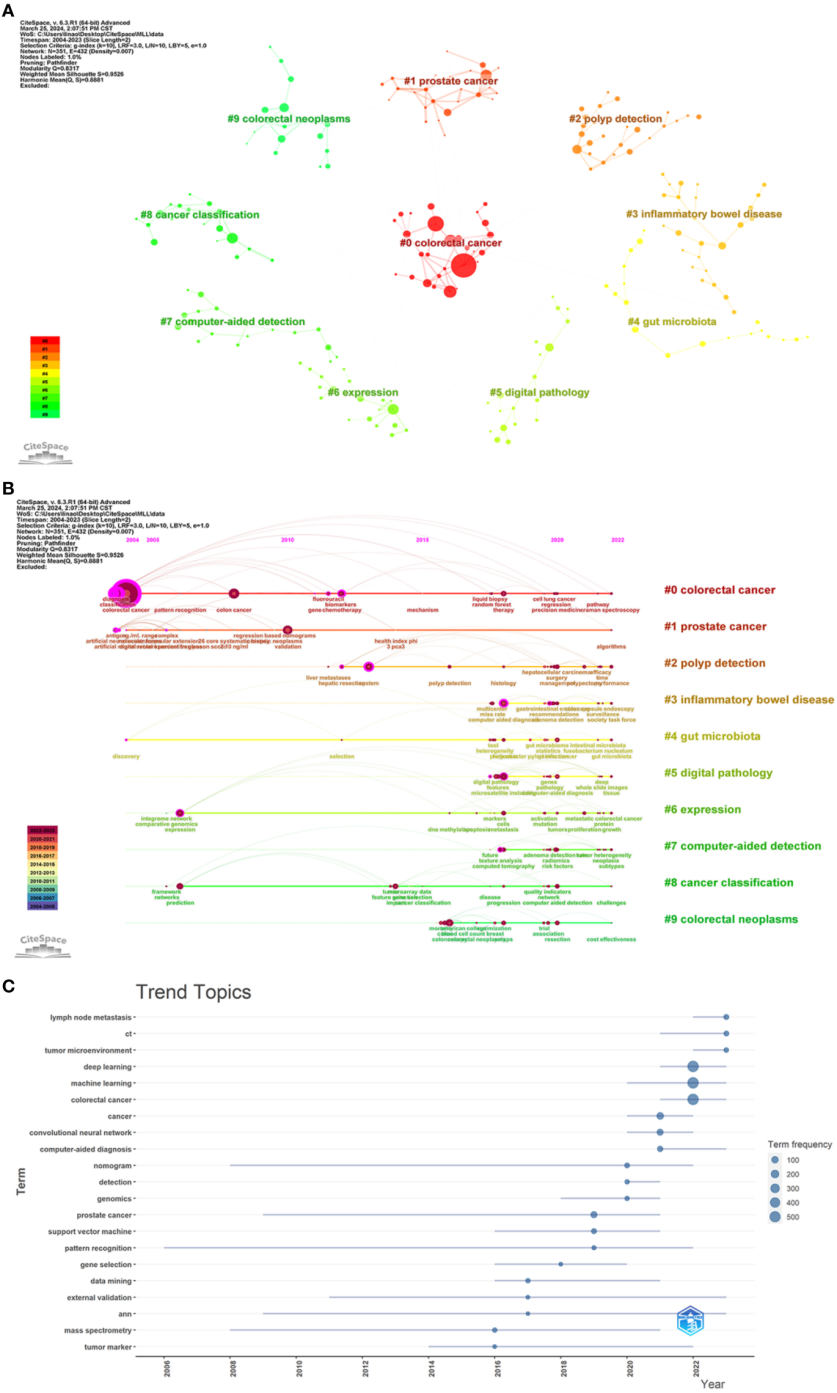
**(A)** Map of keyword clusters. Nodes indicate keywords; a node size indicates how often one keyword appear; a larger node indicates a higher frequency; the number indicates the number of the cluster, with smaller numbers indicating more keywords included in the cluster. **(B)** Timeline graph of keyword clusters. Nodes indicate keywords; the upper position of nodes on the horizontal axis corresponds to the time of the first appearance of the keyword; a larger circle indicates a higher frequency of the keyword; the node color indicates the time of one keyword’s appearance; a color closer to purple indicates an earlier appearance, and a color closer to red indicates a later appearance; the color curve indicates the co-occurrence of keyword clusters of different colors; the cluster tag on the right side represents the theme of the domain. **(C)** Map of thematic trends.

Keywords were clustered by CiteSpace software (**Figure 10A**), revealing the knowledge structure of a particular topic. A Q-value (Modularity) of 0.8317 and an S-value (Silhouette) of 0.9526 indicated that the clusters formed were highly structured and differentiated. Among the top 10 clusters identified (numbered 0-9), cluster #2 focused on polyp detection and covered keywords such as system, optical biopsy, and management; cluster #3 focused on inflammatory bowel disease and contained terms such as endoscopy, confocal laser microendoscopy, capsule endoscopy, and colonoscopy; cluster #5 dealt with digital pathology, with keywords such as microsatellite instability, image analysis, colonoscopy, and panoramic slice images; and cluster #6 focused on gene expression and highlighted the importance of deep learning, artificial intelligence, weighted gene co-expression network analysis, cells, and other concepts; cluster #7 focused on computer-assisted detection, and contained terms such as computed tomography, tumor heterogeneity, texture analysis, etc.; cluster #8 focused on cancer classification, with feature selection, gene expression data, deep learning, and quality metrics as its keywords.

In the CiteSpace, we tracked the evolution of keywords over time by plotting a timeline of keyword clustering (**Figure 10B**). Using the Bibliometrix, we created thematic trend charts based on keywords over the period from 2016 to 2023 (**Figure 10C**). We found that early research focused on CRC diagnosis, classification, and biomarkers, whereas recent research tended to focus on areas such as AI-assisted colorectoscopy, digital pathology, image analysis, gene expression, and prognosis prediction. By analyzing the topic trend map, we found that the hot keywords in current research included lymph node metastasis, computed tomography (CT), and tumor microenvironment, which may guide the direction of future research.

## 4 Discussion

### 4.1 Analysis of the basic situation of the study

This bibliometric analysis reviews the research on AI in CRC diagnosis and treatment in the past 20 years. We found that the number of publications per year grew slowly and never rose above 50 during the period from 2004 to 2016. However, since 2017, this number began to grow significantly, especially between 2020 and 2023, indicating that this research has ushered in a new era since 2017. More encouraging research is expected in the future.

In the world, China ranked first and the USA second in the number of publications, but China sit far below the USA in the total number of citations. In addition, China’s average number of citations was also lower than that of countries such as Sweden, the United States, the Netherlands, the United Kingdom, Canada, and Germany. According to the TSL index, the United States collaborated most frequently with other countries (regions). These data suggest that although China has released the highest number of publications, the overall quality and authority of its research remain to be improved. The United States undoubtedly occupies a central position in this field. High-quality research is mainly contributed by developed countries in Europe and the United States.

At the institutional level, Sun Yat-sen University devoted the highest number of publications, while the Mayo Clinic gained the highest number of citations. Harvard Medical School and Chinese Academy of Sciences ranked high in the number of publications, number of citations, and TSL, demonstrating their heavy contributions in this research area. As for individual contributors, MORI, YUICHI had the highest number of publications for his research on AI-assisted colonoscopy, while WANG, PU was the most cited author for his research on the effect of AI-assisted colonoscopy on adenoma detection. Their contributions were focused on AI-assisted colonoscopy. Finally, based on the total number of citations and impact factor values, the journals “Gastrointest Endosc” and “Gastroenterology” enjoyed the highest reputation in this field.

### 4.2 Current research hotspots

According to the analysis of highly cited studies, co-cited references, and keywords, the current research hotspots are mainly categorized into the following three areas:

#### 4.2.1 Early screening and diagnosis

AI has greatly advanced the development of medical imaging. Features on CT, MRI, and other medical images can be extracted, quantified, and based on to identify and distinguish tumors. AI helps radiologists to detect not only CRC, but also tiny polyps or other early lesions, thus improving diagnostic accuracy and efficiency (33, 34). In colonoscopy, AI can locate and facilitate precise border segmentation of the lesions (24). Deep learning models, such as convolutional neural networks (CNN), can analyze colonoscopy video images in real-time to automatically detect polyps or other abnormal tissues. Some polyps that are small, isochromatic, flat, unclear at borders, hidden by folds, and located at the edges of the field of view, have been easily overlooked by endoscopists, but can now by accurately identified by AI (27, 29, 35). Computer-aided diagnostic (CADx) systems allow optical biopsy and characterization of polyps or other diseased tissues with an accuracy comparable to or higher than that of experienced endoscopists (36, 37).

#### 4.2.2 Pathology and staging

Histopathologic analysis is regarded as the gold standard for assessing cancer diagnosis and prognosis. Whole Slide Imaging (WSI) is being widely around the world used for scanning and digitizing whole tissue sections. AI can extract pathological evidence from these digitized images to support decision-making on lesion type, grading, and other key characteristics (38, 39). To more accurately detect the nuclei, a team of researchers has combined Neighborhood Integration Predictor (NEP) with CNN to quantitate tissue components in the whole slide image (31). Staging of CRC is closely related to lymph node metastasis, lymph nodes with micro-metastases on whole slide images can currently be detected by deep learning models without manual annotation (40). In the future, the application of AI models in this field will be even broader.

#### 4.2.3 Prognostic assessment

An AI model can combine one patient’s clinical information, pathological results, genetic test results, and other multi-dimensional data to assess the prognosis and provide a strategy for personalized treatment (41). Based on images of CRC tissue samples, researchers have combined a CNN with a recurrent architecture to train a deep learning model for predicting CRC prognosis. This model can assess CRC morphology, histopathology, microenvironment more accurately than an experienced physician (42, 43). AI can also automatically extract multi-dimensional prognostic features (such as tumor size, shape, edge) from CT, MRI, and other medical images for prognostic assessment (44). A “meta-model” has been established to assist in clinical decision-making and predict the survival of cancer patients, even their clinical data are incomplete (45). These AI models have substantially sharpened physicians’ ability to predict the outcomes of CRC.

### 4.3 Future research trends

As indicated by the results from the citation analysis and the topic trend analysis, future research of AI in CRC diagnosis and treatment is expected to focus on the following three aspects:

#### 4.3.1 Multimodal data fusion

Most of current AI models are operated in a single paradigm, which limits their application in a broader clinical context. The fusion of paradigms can increase the robustness and accuracy of diagnostic and prognostic AI models. Multimodal fusion of histological images and genomic features has demonstrated higher accuracy in tumor grading and molecular typing than single-peak deep networks trained only on histological and genomic data (46). Faisal Mahmood’s team at Harvard Medical School are exploring the application of AI for multimodal data integration in oncology. Their AI model can discover new associations within and across modalities which can explain variations in outcomes or resistance to treatment, as well as new tumor biomarkers and therapeutic targets (47, 48). AI has demonstrated the ability to analyze complementary multimodal data streams about CRC. Multiorganomics techniques and AI algorithms will become a research hotspot in the future and drive the development of precision medicine for cancer (49).

#### 4.3.2 Personalized treatment and precision medicine

AI can tail out an individualized treatment plan by analyzing a patient’s genetic information, biomarkers, clinical data, and lifestyle. It has shown that AI can predict microsatellite instability (MSI) and defective mismatch repair (dMMR) in CRC patients, thus facilitating the establishment of personalized drug regimens (50). Various algorithms, which can be run on APPs, have been trained to predict a wide range of genetic mutations, molecular tumor subtypes, gene expression signatures, and standard pathological biomarkers in routine pathological tissues (51). With the continuous advancement of AI technology, the treatment of CRC in the future will be more personalized, precise, and efficient.

#### 4.3.3 Drug development

AI can be used to optimize peptide synthesis and molecular design, virtualize molecular docking, quantitate conformational relationships, drug repositioning, protein misfolding, protein-protein interactions, and identify molecular pathways in polypharmacy (52). Using AI, an IncRNA-related ceRNA regulatory network containing 144 core genes has been constructed for CRC, thereby easing the exploration of targeted treatments (53). In summary, AI has aroused a sea change in drug development.

## 5 Limitation

Given that other databases may contain valuable literature, use of only the WoSCC database may have limited the significance of the study. There may have been a language bias from the inclusion of only publications in English, rather than other languages. The quality of selected literature was not assessed, which may have affected the interpretation of the results. Recent high-quality articles were not included into the analysis, which may have discounted the reliability of our findings.

## 6 Conclusion

In the research on AI in CRC diagnosis and treatment, the United States makes a significant contribution by publishing a large number of articles with high qualities and impacts. China also occupies an important position in the volume of published articles, but there is still room for improvement in the overall quality of research. Currently, the main research hotspots are put on early screening and diagnosis (including imaging, colonoscopy, etc.), pathological diagnosis and staging, and prognostic assessment. Future research may turn to multimodal data, personalized treatment, precision medicine, as well as drug development.

## Data Availability

The raw data supporting the conclusions of this article will be made available by the authors, without undue reservation.
